# *Chk1* Haploinsufficiency Results in Anemia and Defective Erythropoiesis

**DOI:** 10.1371/journal.pone.0008581

**Published:** 2010-01-05

**Authors:** Nathan C. Boles, Sirisha Peddibhotla, Alice J. Chen, Margaret A. Goodell, Jeffrey M. Rosen

**Affiliations:** 1 Interdepartmental Program in Cell and Molecular Biology, Baylor College of Medicine, Houston, Texas, United States of America; 2 Department of Pathology, Baylor College of Medicine, Houston, Texas, United States of America; 3 Department of Pediatrics and Center for Cell and Gene Therapy, Baylor College of Medicine, Houston, Texas, United States of America; 4 Department of Molecular and Cellular Biology, Baylor College of Medicine, Houston, Texas, United States of America; Cleveland Clinic, United States of America

## Abstract

**Background:**

Erythropoiesis is a highly regulated and well-characterized developmental process responsible for providing the oxygen transport system of the body. However, few of the mechanisms involved in this process have been elucidated. *Checkpoint Kinase 1* (*Chk1*) is best known for its role in the cell cycle and DNA damage pathways, and it has been shown to play a part in several pathways which when disrupted can lead to anemia.

**Methodology/Principal Findings:**

Here, we show that haploinsufficiency of *Chk1* results in 30% of mice developing anemia within the first year of life. The anemic *Chk1+/−* mice exhibit distorted spleen and bone marrow architecture, and abnormal erythroid progenitors. Furthermore, *Chk1+/−* erythroid progenitors exhibit an increase in spontaneous DNA damage foci and improper contractile actin ring formation resulting in aberrant enucleation during erythropoiesis. A decrease in *Chk1* RNA has also been observed in patients with refractory anemia with excess blasts, further supporting a role for *Chk1* in clinical anemia.

**Conclusions/Significance:**

Clinical trials of Chk1 inhibitors are currently underway to treat cancer, and thus it will be important to track the effects of these drugs on red blood cell development over an extended period. Our results support a role for *Chk1* in maintaining the balance between erythroid progenitors and enucleated erythroid cells during differentiation. We show disruptions in *Chk1* levels can lead to anemia.

## Introduction

**Figure 1:**
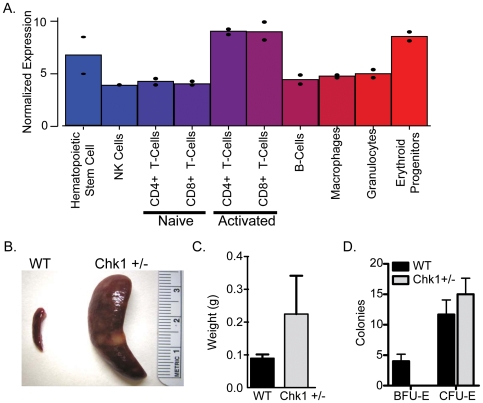
*Chk1* is expressed in erythroid progenitor cells. (A) Expression profile of *Chk1* in the hematopoietic cell types from a previous study [1]. Erythrocytes, granulocytes, and LT-HSCs were isolated from WBM. Erythroid Progenitors were Ter-119^+^, CD3^−^,CD4^−^,CD8^−^,Mac-1^−^,Gr-1^−^, and B220. LT-HSCs were side-population (SP)^+^, Sca-1^+^, c-Kit^+^, and Lin^−^ (Mac-1, Gr-1, Ter119, B220, CD4, CD8) (B) Gross morphology of spleens from a WT and *Chk1* mouse are drastically different. (C) Spleen weights of WT and anemic *Chk1+/−*. (D.) 100,000 WBM cells were plated in Methocult specific for erythroid colony formation. BFU-E and CFU-E were counted 6 days after plating and a lack of BFU-E formation was observed from the *Chk1+/−* cells.

In a recent study, we used microarrays to examine gene expression differences in several hematopoietic cell types and found the cell cycle regulator *Checkpoint Kinase 1 homolog* (*Chk1*) to be highly expressed in hematopoietic stem cells, T-cells, and erythroid progenitors as compared to the other cell types **(** **Figure 1A** **)** [1]. Because *Chk1* deficiency has been shown previously to lead to a block in T-cell differentiation [2], we considered the possibility *Chk1* may play a role in erythropoiesis. Erythroid cell development is a highly regulated multi-stage process resulting in the production of red blood cells (RBCs). Great strides in understanding this process have been made; however many aspects of the path from a hematopoietic progenitor to a mature red blood cell (RBC) remain unclear. The role of erythropoietin in early erythroid differentiation has been well studied. Erythropoietin is the best understood factor promoting erythropoiesis, and is already used in the clinical setting [3]. Yet until recently, the latter phases of erythroid development, i.e. the processes responsible for enucleation and the final stages of differentiation were poorly, if at all, characterized [4], [5], [6], [7].

A wide range of biological pathways have now been shown to contribute to the end-stages of red blood cell (RBC) development beginning with the essential role macrophages play in the enucleation process by engulfing the newly expelled nuclei [6]. Recent and innovative studies done by Ji et. al. have illustrated the roles of two Rho GTPases (*Rac1* and *Rac2*) and *mDia2* in the final steps of red blood cell development. These experiments demonstrated the consequences of failing to properly regulate actin filaments necessary for contractile actin ring (CAR) formation, which is required for erythroblast enucleation to generate mature RBCs [8]. Additional studies have shown the importance of mitochondrial autophagy in the maturation of red blood cells [4]. A role for the retinoblastoma protein (Rb) in erythropoiesis has also been demonstrated, where Rb loss causes a defect in proper RBC differentiation resulting from defects in enucleation and the biosynthesis of mitochondria [5], [9].

*Chk1* is an evolutionarily conserved serine/threonine protein kinase with a known role in genome maintenance and DNA damage response. Chk1 is required for embryonic development and in adult tissues haploinsufficient phenotypes have been observed including increased DNA damage and cell cycle deregulation and has also been suggested to be a candidate tumor suppressor [10]. More recently Chk1's role has expanded into regulating Tousled-like kinases for chromatin remodeling [11], centrosome- and spindle assembly checkpoints [12], [13] and the repression of transcription of the cell cycle proteins cyclin B and Cdk1 [14], [15]. Moreover, we have shown Chk1 plays a vital role in chromosome segregation and cytokinesis durning normal cell division [16]. Thus, Chk1 acts as a critical cell cycle regulator both in the presence and absence of DNA damage.

The human homolog (CHK1) was shown recently in a stressed environment to be necessary for the monoubiquitination of FANCD2, a gene which, when mutated, results in Fanconi anaemia (FA) and is involved in the FA/BRCA pathway [17], [18]. Furthermore, *Chk1* was demonstrated to be necessary for the differentiation of the chronic myelogenous leukemia cell line K562 into erythrocytes after induction with Ara-C [19]. Overall, while *Chk1* is directly or indirectly involved in a diverse set of cellular processes many of its specific roles in development and cell maintenance remain to be uncovered. To determine the function of *Chk1* in erythropoiesis, we took advantage of a *Chk1*+/− mouse (since homozygous nulls are early embryonic lethal) created by the Elledge lab [20]. Here, we demonstrate an increased incidence of anemia in the *Chk1*+/− mice associated with extreme splenomegaly in the affected animals. Disruption of enucleation was observed in the *Chk1*+/− mice, possibly resulting in an increase in erythroid progenitors to compensate. Furthermore, these Chk1+/− erythroid progenitors exhibited a marked increase in spontaneous DNA damage foci and aberrant CAR formation during RBC differentiation. Thus, this study has uncovered a new role for *Chk1* during erythropoiesis and indicates that *Chk1* inhibition can contribute to anemia.

## Results

### *Chk1* haploinsufficiency disrupts cellular architecture of erythropoietic tissues

**Figure 2:**
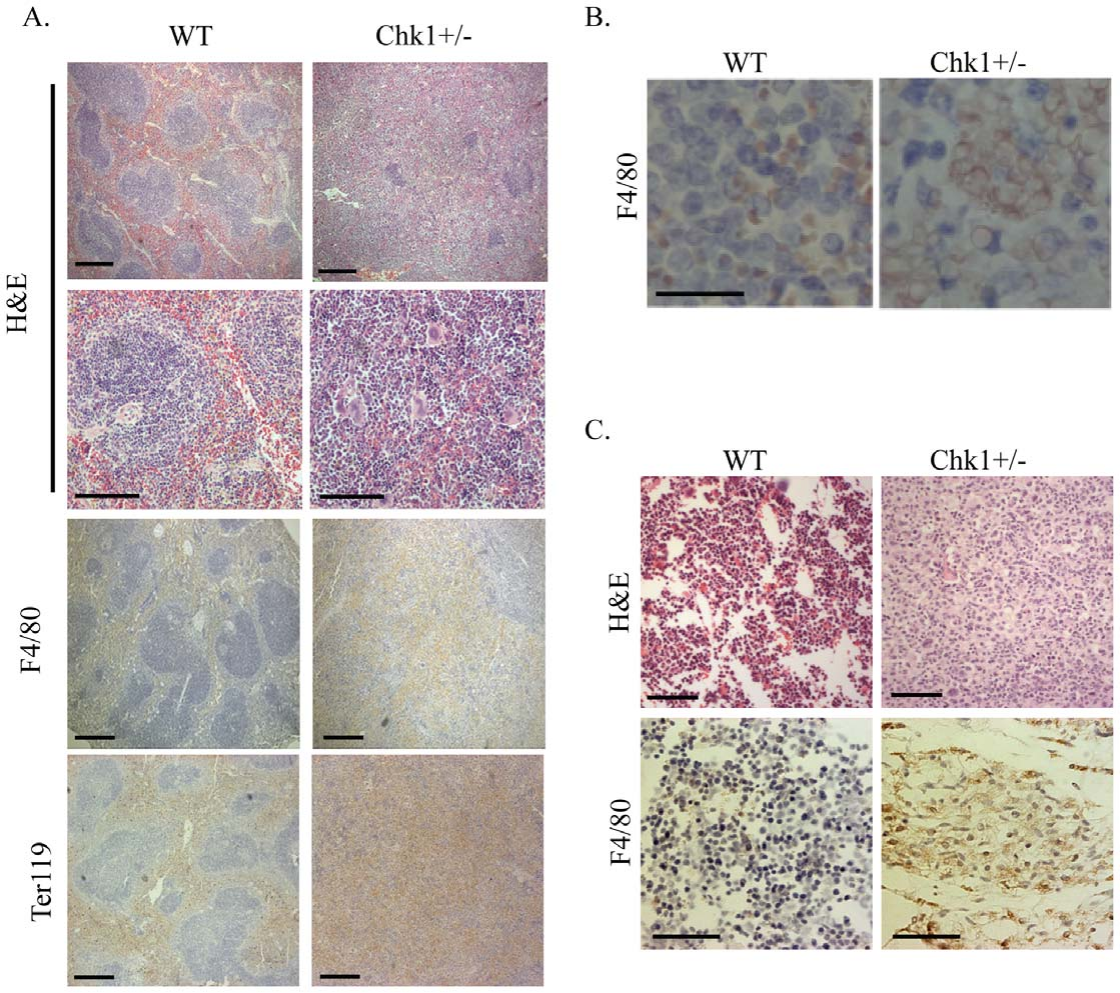
*Chk1*+/− mice show a breakdown of hematopoietic tissue structure with age. (A) H&E stained WT and *Chk1+/−* spleen sections at 5× and 40× magnification and images scaled at 50 µm. Disruption of spleen architecture is plainly visible. Also shown are sections of spleen from a 52-week-old WT and anemic *Chk1*+/− mouse stained with Ter-119 (HPR, brown, 5×) or F4/80 (HPR, brown, 40×) and counterstained with hematoxylin. *Chk1*+/− mice show a breakdown in spleen architecture and a massive increase in histiocytes. (B) WT and *Chk1+/−* spleens stained with F4/80 under 60× magnification and images scaled at 20 µm. (C) H&E sections (20×) of sternum from a WT and *Chk1*+/− mouse showing a loss of bone marrow architecture with a colossal histiocyte invasion. F4/80 (HPR, brown, 40×) stained sternum sections shows an overwhelming increase in histiocytes compared to WT.

We previously used microarray gene expression analysis to identify gene expression differences throughout the hematopoietic cell system. *Chk1* expression was observed in three hematopoietic populations, namely hematopoietic stem cells, activated T-cells, and erythroid progenitors (**Figure 1A**). As defects in T-cell differentiation had already been reported [2], we investigated the effects of losing a *Chk1* allele on erythroid differentiation. Mice heterozygous for *Chk1* exhibit lower levels of Chk1 protein in their spleens (data not shown), which in mice is a major hematopoietic organ. We observed several *Chk1* heterozygous mice with splenomegaly and sudden death at ∼26 weeks of age **(** **Figure 1B & 1C** **)**. Finally, when the Chk1+/− bone marrow cells were plated in Methocult supporting the growth of erythroid colonies we saw a decrease in the number of BFU-E colonies from Chk1+/− marrow compared to WT (**Figure 1D**). However, when cells were plated in Methocult supporting the growth of all hematopoietic colonies the *Chk1+/−* cells performed comparably to WT cells (data not shown). We were intrigued by this observation, since the *Chk1*+/− mice in a mixed genetic background were believed to be phenotypically normal [**20**]. The influence of genetic background in laboratory mouse models has a significant impact on the expected phenotype varying from its viability to behavior. Numerous studies using various mouse models have demonstrated that phenotypic noise can suppress or worsen lethal phenotypes among various genetic backgrounds. Many examples of such mouse models have been widely published such as TGF-β1 –deficient mouse models. Backcrossing of mixed background of TGF-β1-deficient mice to a C57Bl/6 background results in 100% embryonic lethality whereas in a mixed background it is only 50%. This clearly demonstrates the “modifier effect” within various genetic backgrounds in laboratory mouse models that can influence the phenotypic outcome and also variations in the penetrance of the phenotype [21].To identify the cause of the splenomegaly, we performed histopathology of spleens removed from age-matched *Chk1*+/− and wild type (WT) adult mice. Histologic examination of *Chk1*+/− spleens using hematoxylin and eosin staining revealed a range of phenotypes from a mild expansion of red pulp area to an overwhelming disruption of splenic architecture. In the most severe cases, sections of the spleen showed complete loss of normal architecture with markedly expanded red pulp and minimal white pulp. A prominent histocytic infiltrate with evidence of erythrophagocytosis is present, and numerous apoptotic bodies are identifiable (**Figure 2A** **)**. The histocytes, or tissue macrophages, are necessary to engulf apoptotic cells or cell debris and we suspect their incursion into the hematopoietic tissue is a result of the unproductive erythropoiesis and not the cause of the anemia. We used both Ter119, a canonical erythrocyte-specific marker, and F4/80, a macrophage-specific marker, to perform immunohistochemistry on spleen tissue sections from these *Chk1*+/− and WT mice. A remarkable increase in both Ter119 and F4/80 positive staining was observed in the *Chk1*+/− spleen sections when compared to sections from WT counterparts **(** **Figure 2A & 2B** **)**. In summary, affected spleens demonstrate disrupted architecture suggestive of an expanded pool of erythroid precursors with accompanying erythrophagocytosis of defective cells.

Since bone marrow serves as the primary site for red blood cell production, we also examined the bone marrow of *Chk1*+/− mice. Histopathology of the sternum from *Chk1*+/− mice showed near-complete loss of normal hematopoietic elements, replaced almost entirely by a histocytic infiltrate with morphological characteristics similar to that seen in the spleen. Scattered remaining hematopoietic elements show tri-lineage maturation **(** **Figure 2C** **)**. This indicates that *Chk1* haploinsufficiency can lead to serious disruptions of erythropoietic organs such as bone marrow and spleen in mice.

### *Chk1* haploinsufficiency causes anemia in adult mice

**Figure 3:**
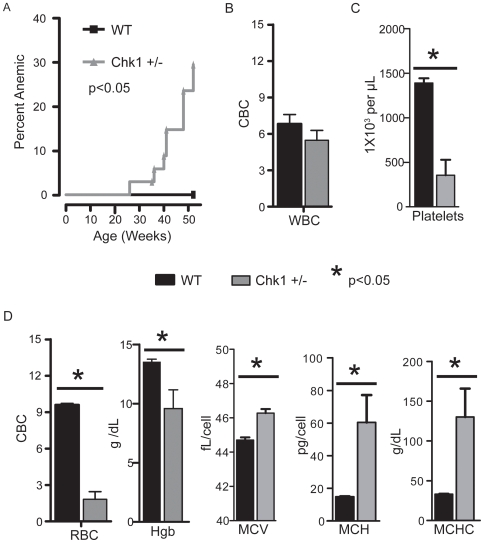
A significant number of *Chk1*+/− mice become anemic with age. (A) Kaplan-Meier plot showing the incidence of anemia in the *Chk1*+/− mice compared to WT mice. A log-rank (Mantel-Cox) test was used to measure significance (p<0.05). Complete blood counts were done on 52-week-old WT and anemic *Chk1*+/− mice and significance was established by a student's t-test. (B) White blood counts (WBC), (C) platelet counts, and (D) red blood cell parameters (red blood cell counts, (RBC), hemoglobin (Hgb), mean corpuscular volume (MCV), mean corpuscular hemoglobin (MCH), and mean corpuscular hemoglobin concentration (MCHC) are shown. A student's t-test was used to determine significance; an asterisk indicates a p<0.05.

Since *Chk1*+/− mice exhibit splenic erythroid imbalance and bone marrow failure, we investigated whether *Chk1* heterozygous mice developed anemia. Studies have shown that chronic erythrocyte depletion in the bone marrow and spleen can cause an impaired stress response and trigger anemia in mice [22]. To conduct these studies, we carefully monitored a large cohort of age-matched WT (n = 24) and *Chk1*+/− mice (n = 34) from 26 weeks of age. The cohort was subjected to complete blood count (CBC) analysis periodically to screen for anemia. Our analysis identified an approximately 30% incidence of severe anemia (decrease in RBC number and hemoglobin count) in *Chk1*+/− mice within 52 weeks of age as compared to no cases of anemia in WT mice over the same time period. Interestingly, while the mean cell volume (MCV) of the *Chk1+/−* erythroid cells was only slightly increased, the mean corpuscular hemoglobin (MCH) and mean corpuscular hemoglobin concentration (MCHC) in these cells was more than tripled indicating the development of a hyperchromic anemia. In addition, we observed significant decreases in platelet counts **(** **Figure 3** **)**. However, we did not observe a significant change in white blood cell number or differential counts between anemic *Chk1*+/− and WT mice. In addition, the severity of anemia did not appear to correlate with increased spleen size in all cases. These results suggest anemia as the most likely cause for the sudden death of *Chk1*+/− mice observed in our mouse colony.

### Miscoordinated erythroid differentiation in *Chk1* haploinsufficient mice

**Figure 4:**
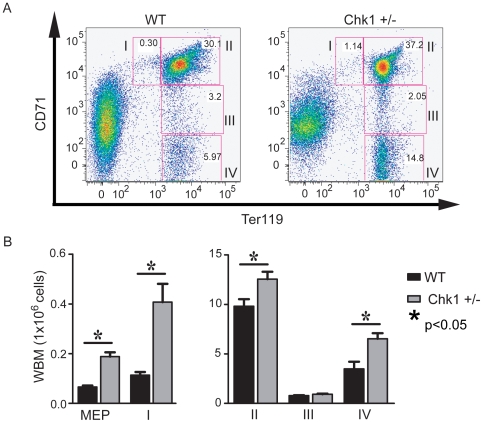
Anemic *Chk1*+/− mice exhibit severe defects in several stages of erythropoiesis. (A) Representative flow cytometry plots showing CD71 and Ter-119 staining used to separate the stages of erythropoiesis for WT and *Chk1*+/− mice. (B) The MEP and stages I–IV of erythropoiesis were analyzed via flow cytometry from the whole bone marrow of anemic *Chk1*+/− mice and 52-week-old WT mice. A student's t-test was used to determine significance; an asterisk indicates a p<0.05.

To understand why *Chk1+/−* mice develop anemia despite their splenic expansion and increasing erythropoiesis, we examined erythroid progenitors of anemic *Chk1*+/− mice and aged matched WT mice. We used immunostaining with Ter119 and CD71 to identify and quantitatively measure the erythroid progenitors in the hematopoietic tissue of anemic *Chk1*+/− mice **(** **Figure 4A** **)** [22], [23]. The Ter119/CD71 immunostaining identifies four cell populations during erythropoiesis, from least differentiated proerythroblasts to more differentiated orthrochromatophilic erythroblasts, and finally giving rise to reticulocytes by enucleation [22], [23]. We observed striking differences between the numbers of erythroid progenitors in the whole bone marrow of WT and anemic *Chk1*+/− mice. The assay revealed a significant increase in the absolute number of early erythroblasts at stage I and II compared to the WT (**Figure 4B**). However, there was no significant difference between the stage III cells of *Chk1*+/− and WT mice. A slight increase at stage IV Ter119^high^ CD71^low^ erythroblasts was also observed in *Chk1*+/− mice compared to WT **(** **Figure 4B** **)**. In addition, we examined the Meg-Erythroid progenitor (MEP) in WT and *Chk1*+/− mice using established markers by flow cytometry, and found a ∼2-fold increase of these progenitors (**Figure 4B**). The increased spleen size and the decrease in the number of viable cells within the spleen may be due to a buildup of apoptotic or malformed cells awaiting engulfment by histocytes. The expansion of early erythroid progenitors and the increased cell death observed in the spleen suggests a block or miscoordination of differentiation at the early erythroblast stage in *Chk1*+/− mice resulting in an increased number of erythroid cells unable to progress through maturation.

### *Chk1+/−* erythroid progenitors show a massive increase in spontaneous DNA damage foci

To understand the cause for aberrant enucleation in *Chk1*+/− erythroid progenitors during erythropoiesis, we used immunostaining of erythroid progenitors with well-studied DNA damage markers pγH2AX and 53BP1. Typically, the DNA damage response pathway is activated in response to DNA double strand breaks(DSBs). This leads to phosphorylation of the histone variant γH2AX by ataxia telangiectasia mutated (ATM) and Rad-3 related ATR kinases, which causes pγH2AX to localize to the site of DSBs. Several other mediator proteins in the DNA damage response pathway such as e.g. MDC1, 53BP1, and BRCA1, are also phosphorylated by ATM/ATR and recruited to the sites of DNA damage [24]. Thus, a complex of damage sensing and repair proteins are assembled at the site of DNA strand breaks to initiate DNA repair and cell cycle arrest through the activation of downstream checkpoint kinases [25].

**Figure 5:**
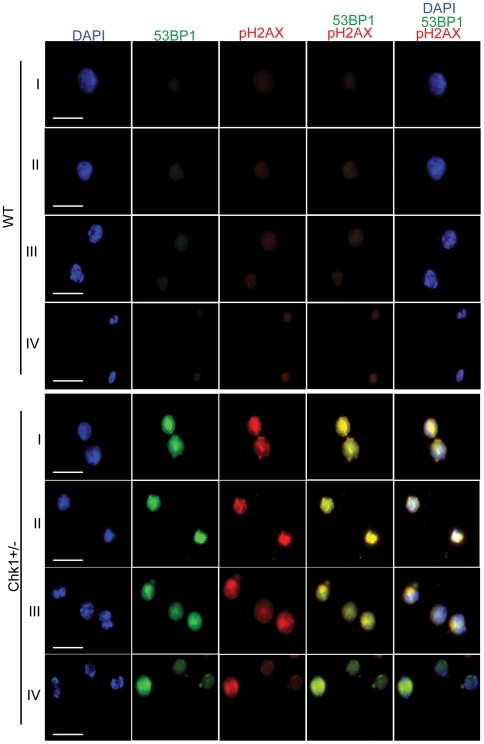
*Chk1+/−* erythroid progenitors show a marked increase in spontaneous DNA damage foci. Progenitors from stages I–IV were sorted from WT and Chk1+/− mice and immunostained with DNA damage markers 53BP1(green), pH2AX(red) and DAPI(blue). The Chk1+/− erythroid progenitors from stages I–IV showed dramatic increase and co-localization of 53BP1 and pH2AX at the site of spontaneous DNA damage and double strand break in the nucleus, resulting in miscoordination of enucleation during erythropoiesis compared to WT counterparts at 60× magnification and images scaled at 20 µm.

Previous studies from our laboratory, demonstrated that proliferating mammary epithelial cells in a conditional *Chk1* heterozygous mouse model showed miscoordinated cell cycle events and spontaneous DNA damage foci formation [10]. Since mammalian erythropoiesis involves proliferation, chromatin changes, and cell-cycle exit to initiate terminal differentiation to mature RBCs, we hypothesized that *Chk1+/−* erythroblasts also might accumulate DNA damage during RBC differentiation. As expected, we observed co-localization of pγH2AX and 53BP1 at the site of DNA damage foci in the nucleus of all the four *Chk1+/−* erythroid populations, from least differentiated stage I to more differentiated stage IV erythroblasts isolated from the bone marrow of young non-anemic *Chk1+/−* mice **(** **Figure 5** **,** **Table 1** **)**. Thus, a marked increase in DNA damage foci was observed in both proliferating and differentiating *Chk1+/−* erythroid progenitors during erythropoiesis, whereas WT erythroid cells did not display DNA damage foci. These results demonstrate that haploinsufficiency of *Chk1* in the proliferating erythroblasts resulted in the accumulation of DNA damage, which possibly caused a block or miscoordinated erythroid differentiation and fewer mature red blood cells.

**Table 1:** Chk1+/− erythroid cells exhibit DNA damage foci.

Genotype	Erythroid Stage	Foci/Cells	% Cells with Foci
Wt	I	1/201	0.5
	II	0/205	0.0
	III	2/200	1.0
	IV	3/210	1.4
		Foci/Cells	% Cells with Foci
Chk1+/−	I	199/200	99.5
	II	200/201	99.5
	III	191/209	91.4
	IV	187/200	93.5

Erythroid cells (Stages I–IV) were isolated via FACS and stained with the DNA damage markers pγH2AX and 53BP1. At least 200 cells per stage were then examined for the formation of DNA damage foci and WT and Chk1+/− mice were then compared. A significant increase in DNA damage foci was observed in the Chk1+/− cells (student's t-test. p<0.001).

### Erythroid progenitors undergo abnormal enucleation during erythropoiesis in *Chk1* haploinsufficient mice

**Figure 6:**
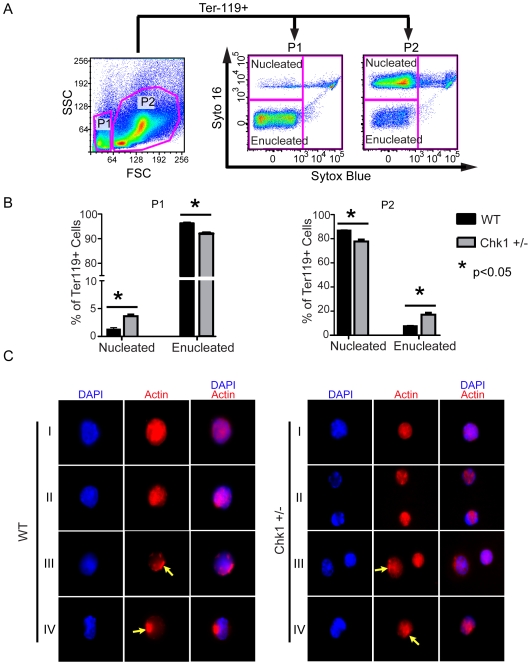
*Chk1*+/− mice show a disruption in enucleation of erythrocytes in the bone marrow. (A) Representative flow cytometry plot of enucleation assay used. Syto 16 is cell permeable, whereas Sytox Blue is cell impermeable, thus the two dyes in tandem allow the separation of enucleated and nucleated erythrocytes [6]. Low FSC Ter-119+ cells or high FSC Ter-119+ were examined for the percentage of nucleated or enucleated cells. Low FSC (small) cells are the more mature erythrocytes, while high FSC (large) cells are the more immature erythrocytes. (B) The P1 gated cells, which should almost entirely consist of RBCs, have fewer properly enucleated erythrocytes with an increase in cells having their nuclei in *Chk1*+/− whole bone marrow. The P2 gated cells, the majority of which should be immature erythrocytes with their nuclei still intact show a decrease in nucleated cells with a concurrent increase in enucleated cells in the *Chk1+/−* whole bone marrow. (C) Progenitors from stages I–IV were sorted from WT and Chk1+/− mice and immunostained with Alexa-flour 594 Phalloidin(red) and DAPI(blue). The WT erythroid progenitors from stages I–IV show dynamics of actin filaments during CAR in erythropoiesis. WT erythroid progenitors in Stages III and IV display formation of CAR (yellow arrows). Aberrant CAR formation (yellow arrows) is observed in stages III and IV *Chk1+/−* erythroid progenitors at 60× magnification and images scaled at 20 µm.

To further investigate the misregulation of erythroid differentiation, we took advantage of a previously developed technique to study enucleation [6]. Briefly, hematopoietic cells are stained with two DNA dyes, one that is impermeable to cell membranes and another which can pass through membranes. Then flow cytometry is used to separate out nucleated cells (only stained by the cell-permeable dye), and enucleated cells (unstained). In our studies, cells were not only examined by size, but by Ter-119 positivity before examining their enucleation status **(** **Figure 6A** **)**. The P1 gated (small) cells should almost entirely consist of RBCs with most cells being enucleated and a few cells having their nucleus. The P2 gated (large) cells should be immature erythrocytes with their nuclei still intact. We examined young (10 weeks old) non-anemic mice to discover if a disruption in erythroid differentiation could be observed before anemia occurred in these mice.

Surprisingly upon examining the P1 gated cells, the young and non-anemic *Chk1*+/− mice exhibited a decrease in enucleated cells, and an increase in nucleated cells as compared to WT mice. The P2 gated Chk1+/− cells showed a significant decrease in nucleated cells and an increase in enucleated cells **(** **Figure 6B** **)**. The increase in inappropriately large enucleated erythroid cells and the decrease in small properly enucleated cells in the *Chk1*+/− mice support the idea of miscoordinated erythroid differentiation. In the *Chk1+/−* mice, a failure of the erythroid progenitors to successfully and appropriately eject their nuclei may result in an influx of histiocytes to dispose of the failed cells.

### *Chk1+/−* erythroid progenitors fail to enucleate during erythropoiesis due to improper CAR formation

The results from the enucleation assay also revealed that *Chk1+/−* erythroid progenitors failed to properly eject their nucleus during the enucleation process. Mammalian erythroid cells enucleate via asymmetric cell division involving extrusion of the condensed nucleus enclosed within a plasma membrane. Numerous studies show that asymmetric cell division in mammals involves cytokinetic components, wherein formation of contractile actin ring at the midbody provides the required momentum for abscission of dividing daughter cells [26]. Past studies have shown that actin filaments also play a critical role in the formation of contractile actin ring (CAR) at the boundary of the cytoplasm of budding reticulocyte and nucleus of enucleating cell [27]. Recent studies have shown CAR formation is indispensable for enucleation and Chk1 appears to interact with the cytokinetic machinery responsible for separating cells during cell division [8], [16], [28].

Therefore, to examine the kinetics and dynamics of actin filaments during CAR formation as shown by Ji et al., we immunostained the four populations of young *Chk1+/−* and WT erythroid cells during erythropoiesis with fluorescently labeled Phallodin, an actin binding protein. Interestingly, we observed aberrant CAR formation in the *Chk1+/−* erythroid cells at stage IV as compared to the CAR observed at stage IV in WT erythroid cells. Moreover, the least differentiated proerythroblasts of *Chk1+/−* mice at stage I also displayed less intense actin staining as compared to the stage I proerythroblasts from WT mice **(** **Figure 6C** **,** **Table 2** **)**. Therefore, these results provide an explanation for the abnormal increase of nucleated P1 gated *Chk1+/−* erythroid cells as compared to WT observed using the enucleation assay. Thus, these results demonstrate that *Chk1* haploinsufficiency may result in improper CAR formation, presumably leading to unsuccessful enculeation during erythropoiesis.

**Table 2:** Chk1+/− show defective CAR formation.

	Total Cells per type	Wt Cells w/defective CAR	Chk1+/− Cells w/defective CAR	Fischer's Exact test
Stage I	300	0	10	P = 0.0017
Stage IV	300	4	59	P<0.0001
Total	600	4	69	P<0.0001

Erythroid cells (Stage I and IV) were examined for defective CAR formation by immunohistiochemistry. Significant differences were calculated by Fisher's exact test.

### *Chk1* expression in human erythroid progenitors and clinical anemia

**Figure 7:**
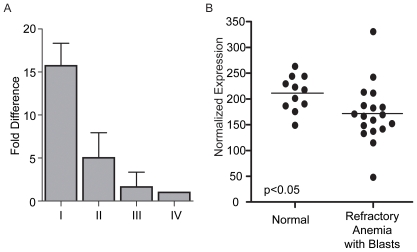
*Chk1* expression in human erythroid progenitors and anemia patients. (A) Taqman real-time PCR was used to examine the expression levels of *Chk1* in human erythroid progenitors and 18s was used as an internal control (n = 3). (B) A decrease in *Chk1* levels (one probe) was observed in patients (individual patients are represented as dots and the graph) with refractory anemia with blasts when compared to healthy individuals (Z-test, p<0.05; GEO DataSets ID = GDS2118; Affy probe ID = 238075_at) [29]. GEO DataSets were searched for the key words anemia and human via the NCBI website (http://www.ncbi.nlm.nih.gov/sites/entrez?db=gds). The results were further filtered to select for studies using hematopoietic progenitors and examining types of anemia. Studies utilizing samples from transplant patients, cancer patients, or malaria patients were discarded. Only one study met these criteria.

To determine whether *Chk1* was also expressed in human erythroid precursors, we obtained cDNA from each stage of erythroid development isolated from human bone marrow and used real time PCR to examine the levels of *Chk1* expression. We found *Chk1* to be highly expressed in stage I progenitors when compared to stage IV cells, and to steadily decrease as the cells differentiated (**Figure 7A**). A similar pattern of expression was observed in the mouse erythroid progenitors **(data not shown)**. Finally, we wanted to determine if a decrease in *Chk1* expression has previously been observed in human anemias. For this we did an *in silico* search for *Chk1* expression in anemias by using GEO Profiles (http://www.ncbi.nlm.nih.gov/geo/). When we examined *Chk1* expression in this data set, we found it significantly decreased in patients suffering from myelodysplastic syndromes with refractory anemia with blasts [29] (**Figure 7B**). These data indicate a possible role for *CHK1* in human erythropoiesis and as a contributor to clinical anemias.

## Discussion

The results presented here show that *Chk1* plays a role in red blood cell formation. A loss of one *Chk1* allele greatly increases the chances of developing anemia in mice and illustrates the importance of maintaining the correct levels of *Chk1* in erythroid progenitor cells. Moreover, a decrease in *Chk1* RNA has been observed in patients with refractory anemia with excess blasts, further supporting an important role for *Chk1* status in clinical anemia [29]. Anemia can occur due to excessive blood loss, death of blood cells, or ineffective blood cell production [30]. Our findings support a role for *Chk1* haploinsufficiency in the latter method of anemia induction.

In many ways the phenotype of the *Chk1+/−* erythroid cells are reminiscent of the erythroid-specific retinoblastoma protein (Rb) knockout cells. This mouse model shows an increase in Ter119+ CD71^high^ cells and a decrease in enucleated cells [5], [9], [31], similar to our *Chk1* mutant mice. However, in the erythroid-specific *Rb*−/− mice, their spleens show an expansion of the red pulp, but not the complete breakdown of splenic architecture observed in the more severely affected *Chk1+/−* mice. In addition, erythroid-specific Rb−/− cells exhibited severe micronucleation and DNA damage, thus affecting enucleation during erythropoiesis [32]. Similarly, we have also observed a marked increase in spontaneous DNA damage foci formation shown by co-localization of DNA damage markers pH2AX and 53BP1 in the nucleus of proliferating and differentiating *Chk1+/−* erythroblasts during erythropoiesis. These studies support the idea that critical cell cycle regulators and checkpoint proteins such as Rb and *Chk1* also play a role in erythroblast enucleation to generate terminally differentiated mature RBCs. Inappropriate expression or regulation of these cell cycle regulators may have a severe impact on the condensing chromatin and cell cycle status of maturing erythroblasts.

Apoptosis has been shown to play a key role in erythropoiesis and *Chk1* has recently been shown to be cleaved by caspases resulting in a hyperactive form of *Chk1* [33], [34]. Caspases have been shown to play key roles in the balance between cell death and fully differentiated red blood cells during erythropoiesis [7], [35]. Furthermore, when siRNA against caspase-3 was expressed in CD34+ progenitor cells, they failed to differentiate into reticulocytes at the same rate as cells transfected with a control siRNA [35] resulting in an increase in the early stage erythroid cells. This phenotype is very similar to what we see in the *Chk1+/−* mice, making this another attractive pathway for future studies.

Finally, studies have shown that erythroid cells evict their nucleus via asymmetric cytokinesis and apparently healthy *Chk1*+/− mice already have small but significant disruptions in their ability to properly enucleate erythroid cells (**Figure 6B**). Our results indicate that improper CAR formation in *Chk1+/−* erythroid cells during late stages of erythropoiesis, provides one of the explanations for unsuccessful enucleation. It is possible that inappropriate regulation of pathways controlling cytokinesis caused by decreased *Chk1* levels might contribute to the improper enucleation of the erythroid cells. The increase in hematopoietic stress of the *Chk1+/−* mice likely leads to signals being sent to the hematopoietic progenitors to increase the number of MEPs in an attempt to compensate for the reduction of normal RBCs. This in turn may increase the amount of failed erythroid cells in the *Chk1+/−* mice possibly resulting in anemia and an even greater influx of histiocytes, which ultimately take over the hematopoietic tissues. Future studies will be required to determine if interactions between *Chk1* and the recently identified adaptor protein mDia2 during CAR formation play any role in enucleation during erythropoiesis.

Ultimately, apparently healthy *Chk1*+/− mice have small but significant disruptions in their ability to properly produce RBCs, as well as additional defects (**Figure S1**), yet only 30% of *Chk1+/−* mice develop anemia. This suggests the existence of a compensatory mechanism that allows the majority of mice to escape full blown anemia. However, not all mice can fully exploit this mechanism, and in these cases yet undefined additional insults to the hematopoietic progenitors appear to occur leading to an unrecoverable defect. Disruptions of DNA repair caused by the decreased dose of *Chk1* could easily lead to additional “hits” in the genome resulting in frank anemia [10]. Thus, we believe *Chk1* haploinsufficiency causes a slight defect in normal erythropoiesis and in the presence of stress. e.g. such as infection, this defect is exacerbated, leading to anemia. Many of *Chk1*'s wide-ranging roles in cellular functions can either directly or indirectly affect proteins and processes already shown to play key roles in erythropoiesis, and it is most likely a combinatorial effect of deficiencies, which individually may exert a relatively minor phenotype, yet together result in anemia in 30% of these mice. Elucidation of the role *Chk1* plays in erythropoiesis may lead to a better understanding of its possible role in clinically relevant anemias, and will be important in designing therapeutic studies in cancer patients using the new generation of Chk1 inhibitors entering clinical trials.

## Materials and Methods

### Mouse colony and aging study

Previously published *Chk1*+/− mice [20] on a mixed genetic background C57Bl/6×129Sv/J were back-crossed for six generations with C57Bl/6 mice (Harlan). Genotyping was carried out by PCR, using mouse tail DNA and specific primers designed against the *Chk1* WT and null allele. The presence of *Chk1* null allele was confirmed by using a primer set against the neomycin gene of the following sequence, NeoF (5′-GAT CGG CCA TTG AAC AAG ATG G-3′) and NeoR (5′-CCT GAT GCT CTT CGT CCA GAT C-3′), respectively.

For the aging study, a large cohort of age-matched *Chk1*+/− and WT mice were pooled at 26 weeks of age and a complete blood count (CBC) was carried out periodically, approximately every 4 weeks. Mice were bled retro-orbitally with EDTA hand-coated capillary tubes and 60 µL of blood was obtained. CBCs were then carried out with either an Abbott Cell-Dyn 3500 or a Drew Scientific Hemavet 950. The mice were sacrificed with an end point of either 52 weeks of age or when they developed anemia (an RBC count below normal range). Mice used for all these studies were euthanized according to IACUC approved animal protocol guidelines.

### Flow cytometry of erythroid progenitors

Whole bone marrow and spleen was isolated, made into single cell suspensions, and counted with a Vi-Cell (Beckman Coulter) and trypan blue exclusion was used to calculate viability. Cells were taken to a concentration of 100 million cells per mL. They were then stained on ice with various antibody cocktails to identify each progenitor compartment as previously described [22], [36] (all antibodies were obtained from BD PharMingen, San Diego, CA and used at a concentration of 1∶100 unless otherwise indicated). MEP were stained with biotin Lineage markers (gr-1, ter-119, CD4, CD8, CD3, B220, CD19), IL7ra PE-Cy7 (eBioscience), Sca-1 FITC, and cKit PE, CD34 Alexa Fluora 647(at 1∶50)(eBioscience), and CD16/32 Alexa Fluor 700 (at 1∶50)(eBioscience) for 20 min. Cells were then spun down, resuspended, and stained with strepavidin-Pacific Blue(1∶50) for 20 min. Stage I–IV erythroid cells were stained with Ter-119-APC and CD71-PE for 20 min. Finally, cells were then spun down, resuspended in a propidium iodide solution and analysis was accomplished on live cells with an LSRII (Becton Dickinson, Franklin Lakes, NJ).

Human erythroid progenitors were isolated in a fashion similar to mouse cells, however glycophorin A was used instead of Ter-119 since there is no known human Ter-119. Ter-119 is associated with cell surface glycophorin A, a well-known erythroid marker for humans, making it a reasonable substitute [37].

### Tissue preparation and immunohistochemistry

Spleen and sternum from *Chk1* heterozygous and WT adult mice were isolated and fixed overnight in 4% paraformaldehyde (PFA) and stored in 70% ethanol at 4°C before subjecting to further processing. The specimens were paraffin-embedded and 5 µm serial sections were cut on Probe on Plus slides (Fisher Scientific). For histology, these sections were deparaffinized with xylene, and rehydrated with varying concentration of ethanol. This was followed by hematoxylin staining for 5 min and a quick rinse in acid ethanol. Finally, the sections were immersed in eosin stain for 45 sec and then dehydrated with ethanol and xylene overnight. They were mounted with Permount and air-dried completely before imaging under Zeiss Axioplan II upright microscope.

For immunohistochemistry, similar deparaffinization was performed with xylene and ethanol and followed by heat-induced antigen retrieval using 10 mM sodium citrate by boiling for 20 min. All washes were performed with 1× PBS throughout the procedure unless otherwise stated. Sections were then incubated with the respective antibodies Ter119 (1∶2000) or F4/80 (1∶10,000) in 5% BSA and 0.5% blocking buffer. Sections were washed and incubated with anti-mouse (Oncogene Research) or anti-rat (Oncogene Research) secondary antibodies diluted 1∶1000 in 5% BSA and 0.5% blocking buffer for 1 hour at RT. Vectastain Elite ABC and diaminobenzidine (DAB) substrate kits were used to detect immunoperoxidase staining according to the manufacturer's instructions (Vector Laboratories). Slides were counterstained with hematoxylin for 30 sec, dehydrated, and mounted using Permount. The sections were air-dried completely and imaged using a Zeiss Axioplan II upright microscope. All pictures were scaled to size for figures with Adobe Illustrator.

#### Immunostaining and microscopy

After isolation by FACS, erythroid cells from Chk1+/− and WT mice bone marrow were applied on to the slides by placing a drop of 10,000 cells on each slide and allowing them to air dry to the slide. The cells were fixed with 4%PFA, permeabilized with 0.5% Triton X-100 and incubated with 5% goat serum (Sigma, St. Louis, MO) mixed with primary antibody at dilutions of anti-pγH2AX (Upstate, CA) 1∶500 and anti-53BP1(Bethyl laboratories, CA) at 4C overnight. All the cells were washed 3 times with 1× PBS for 10 min and incubated with 5% goat serum mixed with respective fluorochrome-conjugated secondary antibodies or Alexa flour 594 Phalloidin(Invitrogen) at 1∶1500 for 1 hr at RT. Cells are washed 5 times for 15 min and counterstained with Vectashield 4,6-diamidino-2-phenylindole (DAPI) to visualize DNA. Microscopic analysis was performed using an Olympus BX50 Fluoresence microscope (Applied Precision, Washington) at 60× magnification using 2×2 bin and images were processed using Spot software and Adobe Photoshop.

### Erythroid Colony Formation

Whole bone marrow (WBM) was collected from WT and *Chk1+/−* mice and 100,000 WBM cells were plated onto Methocult M3436 (Stem Cell Technologies), which drives and supports formation of BFU-E and CFU-E colonies only, in six well plates. Six days after plating cells, colonies were counted and typed.

### Real-Time PCR

RNA was isolated from 1×10^5^ cells (except for HSCs which was from 2.5–5×10^4^ cells), using the RNAqeuous kit (Ambion, Austin, TX, USA), treated with DNAseI, and precipitated with phenol∶chloroform∶isoamyl alcohol. RNA was quantified using a Nanodrop and 30 ng of RNA was used for each RT-PCR reaction. RT-PCR was performed with Superscript II (Invitrogen). Taqman master mix and *CHK1* taqman probe (Applied Biosystems) was used for the real-time PCR reactions and 18s was used as an internal control. All reactions were run on an ABI prism 7900HT according to manufacturer's protocol (Applied Biosystems).

### Enucleation Assay

Whole bone marrow cells were isolated, made into single cell suspensions, and stained on ice with Ter119-APC (BD PharMingen, San Diego, CA (1∶100)) for 20 min. Cells were then spun down and resuspended with 10 µM Syto16 (Invitrogen) and 8 µM Sytox Blue (Invitrogen) in HBBS with 10% FBS. Cells were allowed to sit at room temperature for 20 min; they were then analyzed with an LSRII (Becton Dickinson, Franklin Lakes, NJ).

### Data Mining

An *in silico* search for *Chk1* expression in anemias by using GEO Profiles (http://www.ncbi.nlm.nih.gov/geo/). GEO DataSets were searched for the key words anemia and human via the NCBI website. The results were further filtered to select for studies using hematopoietic progenitors and examining types of anemia. Studies utilizing samples from transplant patients, cancer patients, or malaria patients were discarded. Finally, the normalized expression data from the GEO profile was shown and a Z-test was used to test for differences.

### Statistical Analysis

All statistical analyses was carried out with GraphPad Prism version 5.00 (GraphPad Software, San Diego California USA, www.graphpad.com). The incidence curve was plotted by the Kaplan-Meier method and the p value was calculated by the log-rank (Mantel-Cox) test. All other p values reported are derived by a student's t-test unless otherwise indicated. Microsoft Excel was used for the performance of Z-test.

## Supporting Information

Figure S1 Non-anemic Chk1+/− mice at 52 weeks show differences form WT mice. Data collected from the non-anemic Chk1+ show differences in the numbers of progenitors of these mice when compared to WT or anemic Chk1+/− mice. However, WBC, RBC, HGB, HCT, and platelet counts in the non-anemic Chk1+/− mice are the same as WT mice. (A) In both anemic and non-anemic Chk1+/− mice the numbers of MEP (CD34− CD16/32− cKit+ Sca1− Lin− Il7ra−) are elevated. However, in the non-anemic Chk1+/− the numbers of GMP (CD34+ CD16/32+ cKit+ Sca1− Lin− Il7ra−) and CMP (CD34+ CD16/32− cKit+ Sca1− Lin− Il7ra−) are also increased, whereas in the anemic Chk1+/− the numbers of CMP are similar to WT and the numbers of GMP are decreased when compared to WT. (B) The non-anemic Chk1+/− mice show a similar increase in the numbers of erythroid cells in Stages I, II, and IV as anemic Chk1+/− mice. However, the numbers of each stage of erythroid progenitors are not significantly increased when compared to WT erythroid progenitor numbers.
(0.43 MB TIF)

## References

[1] Chambers SM, Boles NC, Lin KY, Tierney MP, Bowman TV (2007). Hematopoietic Fingerprints: An Expression Database of Stem Cells and Their Progeny.. Cell Stem Cell.

[2] Zaugg K, Su YW, Reilly PT, Moolani Y, Cheung CC (2007). Cross-talk between Chk1 and Chk2 in double-mutant thymocytes.. Proc Natl Acad Sci U S A.

[3] Koury MJ, Sawyer ST, Brandt SJ (2002). New insights into erythropoiesis.. Curr Opin Hematol.

[4] Sandoval H, Thiagarajan P, Dasgupta SK, Schumacher A, Prchal JT (2008). Essential role for Nix in autophagic maturation of erythroid cells.. Nature.

[5] Sankaran VG, Orkin SH, Walkley CR (2008). Rb intrinsically promotes erythropoiesis by coupling cell cycle exit with mitochondrial biogenesis.. Genes Dev.

[6] Yoshida H, Kawane K, Koike M, Mori Y, Uchiyama Y (2005). Phosphatidylserine-dependent engulfment by macrophages of nuclei from erythroid precursor cells.. Nature.

[7] Zermati Y, Garrido C, Amsellem S, Fishelson S, Bouscary D (2001). Caspase activation is required for terminal erythroid differentiation.. J Exp Med.

[8] Ji P, Jayapal SR, Lodish HF (2008). Enucleation of cultured mouse fetal erythroblasts requires Rac GTPases and mDia2.. Nat Cell Biol.

[9] Clark AJ, Doyle KM, Humbert PO (2004). Cell-intrinsic requirement for pRb in erythropoiesis.. Blood.

[10] Lam MH, Liu Q, Elledge SJ, Rosen JM (2004). Chk1 is haploinsufficient for multiple functions critical to tumor suppression.. Cancer Cell.

[11] Groth A, Lukas J, Nigg EA, Sillje HH, Wernstedt C (2003). Human Tousled like kinases are targeted by an ATM- and Chk1-dependent DNA damage checkpoint.. Embo J.

[12] Kramer A, Mailand N, Lukas C, Syljuasen RG, Wilkinson CJ (2004). Centrosome-associated Chk1 prevents premature activation of cyclin-B-Cdk1 kinase.. Nat Cell Biol.

[13] Zachos G, Black EJ, Walker M, Scott MT, Vagnarelli P (2007). Chk1 is required for spindle checkpoint function.. Dev Cell.

[14] Shimada M, Niida H, Zineldeen DH, Tagami H, Tanaka M (2008). Chk1 is a histone H3 threonine 11 kinase that regulates DNA damage-induced transcriptional repression.. Cell.

[15] Shimada M, Nakanishi M (2008). Checkpoints meet the transcription at a novel histone milestone (H3-T11).. Cell Cycle.

[16] Peddibhotla S, Lam MH, Gonzalez-Rimbau M, Rosen JM (2009). The DNA-damage effector checkpoint kinase 1 is essential for chromosome segregation and cytokinesis.. Proc Natl Acad Sci U S A.

[17] Guervilly JH, Mace-Aime G, Rosselli F (2008). Loss of CHK1 function impedes DNA damage-induced FANCD2 monoubiquitination but normalizes the abnormal G2 arrest in Fanconi anemia.. Hum Mol Genet.

[18] Enders GH (2008). Expanded roles for Chk1 in genome maintenance.. J Biol Chem.

[19] Takagaki K, Katsuma S, Kaminishi Y, Horio T, Tanaka T (2005). Role of Chk1 and Chk2 in Ara-C-induced differentiation of human leukemia K562 cells.. Genes Cells.

[20] Liu Q, Guntuku S, Cui XS, Matsuoka S, Cortez D (2000). Chk1 is an essential kinase that is regulated by Atr and required for the G(2)/M DNA damage checkpoint.. Genes Dev.

[21] Doetschman T (2009). Influence of genetic background on genetically engineered mouse phenotypes.. Methods Mol Biol.

[22] Socolovsky M, Nam H, Fleming MD, Haase VH, Brugnara C (2001). Ineffective erythropoiesis in Stat5a(−/−)5b(−/−) mice due to decreased survival of early erythroblasts.. Blood.

[23] Socolovsky M (2007). Molecular insights into stress erythropoiesis.. Curr Opin Hematol.

[24] Adams MM, Carpenter PB (2006). Tying the loose ends together in DNA double strand break repair with 53BP1.. Cell Div.

[25] Motoyama N, Naka K (2004). DNA damage tumor suppressor genes and genomic instability.. Curr Opin Genet Dev.

[26] Skop AR, Liu H, Yates J, Meyer BJ, Heald R (2004). Dissection of the mammalian midbody proteome reveals conserved cytokinesis mechanisms.. Science.

[27] Takano-Ohmuro H, Mukaida M, Morioka K (1996). Distribution of actin, myosin, and spectrin during enucleation in erythroid cells of hamster embryo.. Cell Motil Cytoskeleton.

[28] Peddibhotla S, Rosen JM (2009). Chking and executing cell division to prevent genomic instability.. Cell Cycle.

[29] Pellagatti A, Cazzola M, Giagounidis AA, Malcovati L, Porta MG (2006). Gene expression profiles of CD34+ cells in myelodysplastic syndromes: involvement of interferon-stimulated genes and correlation to FAB subtype and karyotype.. Blood.

[30] Kruse A, Uehlinger DE, Gotch F, Kotanko P, Levin NW (2008). Red blood cell lifespan, erythropoiesis and hemoglobin control.. Contrib Nephrol.

[31] Spike BT, Dirlam A, Dibling BC, Marvin J, Williams BO (2004). The Rb tumor suppressor is required for stress erythropoiesis.. Embo J.

[32] Dirlam A, Spike BT, Macleod KF (2007). Deregulated E2f-2 underlies cell cycle and maturation defects in retinoblastoma null erythroblasts.. Mol Cell Biol.

[33] Matsuura K, Wakasugi M, Yamashita K, Matsunaga T (2008). Cleavage-mediated activation of Chk1 during apoptosis.. J Biol Chem.

[34] Peller S, Frenkel J, Lapidot T, Kahn J, Rahimi-Levene N (2003). The onset of p53-dependent apoptosis plays a role in terminal differentiation of human normoblasts.. Oncogene.

[35] Carlile GW, Smith DH, Wiedmann M (2004). Caspase-3 has a nonapoptotic function in erythroid maturation.. Blood.

[36] Challen G, Boles NC, Lin KY, Goodell MA (2008). Mouse Hematopoietic Stem Cell Identification And Analysis.. Cytometry.

[37] Kina T, Ikuta K, Takayama E, Wada K, Majumdar AS (2000). The monoclonal antibody TER-119 recognizes a molecule associated with glycophorin A and specifically marks the late stages of murine erythroid lineage.. Br J Haematol.

