# Supplementary figures and images for: Chk1 Haploinsufficiency Results in Anemia and Defective Erythropoiesis
Source: PLoS One. 2010 Jan 5;5(1):e8581. doi: 10.1371/journal.pone.0008581 (PMC2798715; doi:10.1371/journal.pone.0008581)

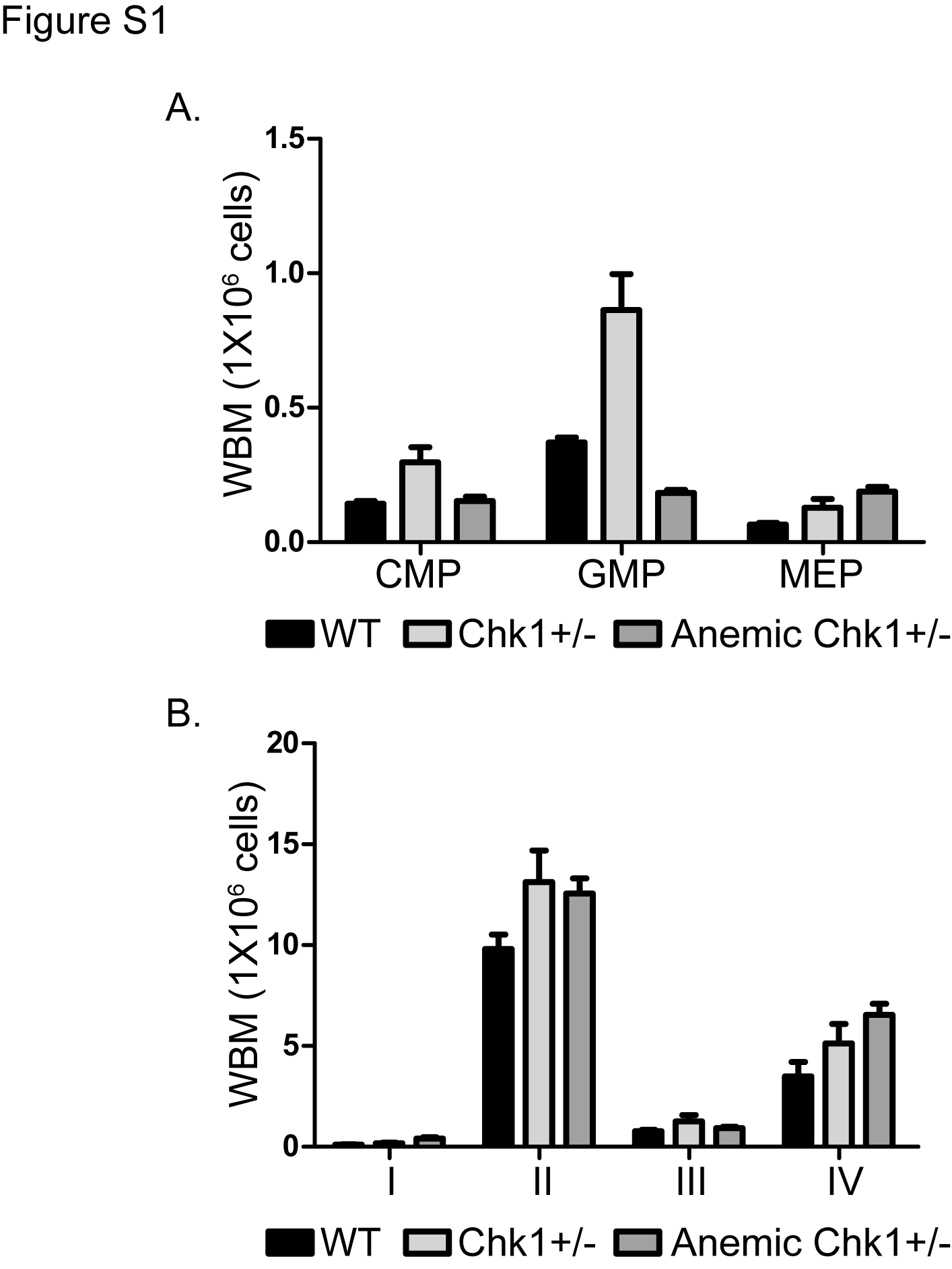

Supplement: Figure S1 — Non-anemic Chk1+/− mice at 52 weeks show differences form WT mice. Data collected from the non-anemic Chk1+ show differences in the numbers of progenitors of these mice when compared to WT or anemic Chk1+/− mice. However, WBC, RBC, HGB, HCT, and platelet counts in the non-anemic Chk1+/− mice are the same as WT mice. (A) In both anemic and non-anemic Chk1+/− mice the numbers of MEP (CD34− CD16/32− cKit+ Sca1− Lin− Il7ra−) are elevated. However, in the non-anemic Chk1+/− the numbers of GMP (CD34+ CD16/32+ cKit+ Sca1− Lin− Il7ra−) and CMP (CD34+ CD16/32− cKit+ Sca1− Lin− Il7ra−) are also increased, whereas in the anemic Chk1+/− the numbers of CMP are similar to WT and the numbers of GMP are decreased when compared to WT. (B) The non-anemic Chk1+/− mice show a similar increase in the numbers of erythroid cells in Stages I, II, and IV as anemic Chk1+/− mice. However, the numbers of each stage of erythroid progenitors are not significantly increased when compared to WT erythroid progenitor numbers. (0.43 MB TIF) [file pone.0008581.s001.tif]
